# Prospects for antibiotic-free poultry production in South Africa: An analysis of the enablers and stumbling blocks

**DOI:** 10.1016/j.onehlt.2025.101144

**Published:** 2025-07-19

**Authors:** Xola Nduku, Thuthuzelwa Stempa, Nobuhle Sharon Lungu, Tandile Nwabisa Ndobeni, Victor Mlambo

**Affiliations:** a University of Mpumalanga, Cnr R40 and D725 Roads, Mbombela 1200, South Africa; b ASCEND, African Climate and Development Initiative, University of Cape Town, Cape Town 7701, South Africa; c University of South Africa, 28 Pioneer Ave, Florida Park, Roodepoort 1709, South Africa

**Keywords:** Antimicrobial resistance, Antibiotic growth promoters, Antibiotic-free poultry production, Regulatory framework, Sustainable alternatives

## Abstract

For decades, antibiotics have been widely used in intensive poultry production to enhance bird health, welfare, growth performance, and product safety. In South Africa, a significant proportion of antibiotics produced is used for growth promotion, contributing to the profitability of intensive poultry systems. Indeed, the country has recently been identified as one of the major contributors to the global increase in antibiotic use. While antibiotics play a crucial role in maintaining bird health, their subtherapeutic use as growth promoters contributes to antimicrobial resistance (AMR) in pathogens as well as unacceptable levels of antibiotic residues in poultry products. Because of their negative impacts on the health of both humans and animals, antibiotic growth promoters (AGP) have been banned or restricted in some countries. Despite the global trend towards AGP-free poultry production, several developing countries, including South Africa, continue to rely on AGPs. The reluctance to transition to AGP-free systems is linked to stumbling blocks such as a lack of sustainable alternatives, higher disease burdens in tropical and subtropical regions, lower biosecurity, and inadequate regulatory and monitoring frameworks. Despite these challenges, there exists an impressive array of practices, technologies, and alternative antimicrobials that could be exploited to drive the transition to AGP-free poultry production. In this review, we examine the current state of AGP use in South African poultry, its role in fostering AMR, and the effectiveness of regulatory measures in curbing inappropriate antibiotic use. By exploring the stumbling blocks and enablers, the review provides a platform upon which the transition to AGP-free poultry production can be built in South Africa and other countries facing similar challenges.

## 1 Introduction

Traditionally, antibiotics have been utilized to mitigate stress-induced maladies in intensively reared poultry, thereby enhancing bird health, welfare, and growth as well as ensuring product safety. For example, Eagar et al. [1] reported that 66 % of the 1500 tons of antibiotics produced between 2002 and 2004 were used as growth promoters in food animals in South Africa. Since then, global antibiotic use in food animals has risen from an estimated 63,200 tons in 2010 to a projected 105,600 tons by 2030 [2], driven by growing demand for poultry products. However, this practice has recently come under intense scrutiny due to its significant role in driving antimicrobial resistance (AMR) [3,4]. This growing public health issue has spurred global initiatives to regulate and reduce the use of antibiotics for growth promotion in livestock. Using antibiotics responsibly, along with strong surveillance and monitoring systems to enforce regulations, is essential to preventing the emergence and spread of antibiotic-resistant bacteria [5]. In response to these global efforts, the European Union banned antibiotics as growth promoters in 2006, leading a trend that has since been adopted in other regions, with China implementing a similar ban in 2020 [6,7]. However, most developing countries, including South Africa, have yet to impose such restrictions on antibiotic growth promoters [8].

The contribution of antibiotic growth promoters (AGP) to the profitability of intensive poultry production in developing countries cannot be overstated. Antibiotic growth promoters have a positive impact on gut microbes, nutrient absorption, and immune function of birds [9]. In addition, there is no doubt that the use of antibiotics for therapeutic purposes in poultry production has been essential for promoting bird health and welfare, as well as ensuring the safety of consumed products in intensive production systems. This is despite antibiotic use in food animals being identified as a major driver of antimicrobial resistance in key pathogens that affect both humans and animals [2,9,10]. Most developing countries have shown a reluctance to transition to AGP-free poultry production due to several challenges [11,12]. These include higher disease burdens and lower biosecurity systems prevalent in some of the developing countries located in the tropics and subtropics. Another significant barrier is the lack of viable, sustainable alternatives to AGPs. Additionally, existing regulations and policy frameworks in South Africa and other developing countries may be insufficient to drive the transition to AGP-free poultry production. Carefully crafted regulations and policies can play a key role in promoting the shift to AGP-free poultry production through the implementation of gradual AGP bans/restrictions, creating incentives, setting biosecurity standards, subsidizing AGP alternatives, creating a certification program and market incentives, and promoting research and innovation.

This review explores the current state of AGP use in South African poultry and its role in driving antimicrobial resistance, which leads to poor health outcomes for both humans and animals. We also assess the effectiveness of the country's regulatory and monitoring frameworks in ensuring appropriate antibiotic use. The review analyzes the obstacles that must be overcome and the opportunities that should be leveraged to facilitate the transition to AGP-free poultry production. Finally, we discuss the prospects for phasing out AGPs in favor of sustainable alternatives, such as prebiotics, probiotics, enzymes, organic acids, and phytochemicals.

## 2 Global use of antibiotic growth promoters

For over half a century, antibiotics have been used to maximize nutrient utilization for growth promotion and productivity in food-producing animals, including poultry [13,14]. The AGP effect was discovered in the 1940s, when beneficial effects on production efficiency in poultry and swine were observed [15]. Since then, AGPs have been included in animal feed at subtherapeutic doses for the prevention of bacterial infections to enhance growth rate and feed utilization efficiency [13,16]. The use of antibiotics has been projected to rise due to increased adoption of large-scale intensive farming operations in countries such as Brazil, Russia, India, China, and South Africa [17]. In a study by Van Boeckel et al. [2], the global consumption of antibiotics in food animal production was estimated at approximately 63,200 tons in 2010 and projected to rise by 67 % (105,600 tons) by 2030. The anticipated increase in antibiotic consumption is due to the growing number of animals raised intensively for food production. However, these projections predate recent global efforts to restrict or ban AGPs. With many high-income countries adopting AGP-free systems, actual global antibiotic use may be lower than earlier estimates. However, in many low- and middle-income countries, including South Africa, where AGP regulations are limited or weakly enforced, antibiotic use in intensive livestock production remains prevalent and may continue to rise without effective policy intervention. The impact of AGP has been particularly important for the poultry industry, which has expanded more than any other animal protein source over the past half-century [18]. Like in most developing countries, South African poultry producers are also transitioning to highly cost-effective and intensive production systems, which rely on antibiotics to optimize health and productivity [19]. Furthermore, the use of antibiotics in the food production chain is generally viewed as crucial for maintaining a consistent supply of healthy animals, thereby enhancing profitability, production efficiency [20], and food safety. However, the emergence of antibiotic-resistant human pathogens and disruptions to the natural gut microbiota led to the phased withdrawal and eventual ban of AGPs in animal feed within the European Union (EU) in 2006 [9,10,13,21]. The growing pressure to prohibit the use of these antibiotics stems from concerns that they may induce cross-resistance in pathogenic bacterial strains that can affect humans [22,23]. Although the EU has established an AGP-free market, many food animal producers in other countries have been slow to transition away from AGP use. For example, South Africa has not adopted any measures to phase out the use of AGPs in animal feeds [11]. Indeed, intensively reared poultry and pigs consume the highest volumes of antibiotics, followed by feedlot cattle and dairy cows in South Africa [24]. However, to minimize antibiotic residues, poultry are typically fed an AGP-free withdrawal diet prior to slaughter, allowing for clearance of AGPs from muscle tissue [25].

## 3 Antibiotic use in South African poultry farms

In South Africa, antibiotics approved for animal growth promotion include virginiamycin, josamycin, flavophospholipol, and poly 2-propenal 2-propenoic acid, compounds that are banned in the EU [24,26]. Additionally, van den Honert et al. [4] reported that penicillin, erythromycin, tetracycline, and sulfonamides are frequently used both for growth promotion and for treating staphylococcal and other infections in food-producing animals. Although these compounds vary in their antimicrobial spectrum and antibacterial mechanisms, it is still unclear whether they enhance growth performance through similar or distinct pathways [27].

In South Africa and most developing countries, records on the sales and use of antibiotics in animal production are scarce, making it difficult to identify trends and patterns of antibiotic consumption [17,28]. Eagar et al. [1] analyzed antibiotic sales data (2002–2004) from eight pharmaceutical companies for use in food-producing animals and reported an average annual sale of 1,538,443 kg of active compounds, primarily macrolides (42.4 %) and tetracyclines (16.7 %). A report published by the Department of Health (DoH) in 2018 showed that antibiotic sales for animal use increased by 58 % from 1,005,763 kg in 2014 to 1,592,842 kg in 2015 [29]. Currently, SA lacks an effective antibiotic use monitoring framework at the farm and species-level [11,29]. Such a framework could be based on on-farm records and financial data to quantify antibiotic purchases and utilization at the farm level. Implementing this approach, however, would be difficult for subsistence farmers, as they typically have inadequate record-keeping practices [17].

## 4 Factors driving antibiotic use

The intensification of animal agriculture in response to growing consumer demand for affordable animal protein perpetuates AGP use in most developing countries, including South Africa. In the South African poultry industry, AGPs are widely utilized to enhance animal productivity and profitability, especially under intensive production systems where overcrowding and suboptimal environmental conditions can induce stress-related diseases [30]. In such environments, the risk of disease outbreaks is higher, requiring subtherapeutic doses of antibiotics to prevent infections, reduce morbidity and mortality rates, and maintain bird health [31,32]. The poultry sector in South Africa operates on narrow profit margins and faces significant challenges, including competition from cheap poultry imports and the absence of government subsidies. As a result, an immediate ban on AGPs could have short- to medium-term adverse effects on food security in the region [19]. All these factors are important in driving the use of AGPs by farmers, who are primarily motivated by profit.

On the other hand, pharmaceutical companies and feed manufacturers face minimal regulatory constraints with regard to production, marketing, or use of in-feed AGPs for food animals. The lack of a robust regulatory framework creates an enabling environment for the wanton production and use of antibiotics in food animals [24]. Feed manufacturers may also avoid scrutiny by protecting their feed formulations, including those incorporating AGPs, as trade secrets. This practice, designed to safeguard feed manufacturers' competitive edge, inadvertently contributes to a lack of transparency regarding the ingredients of commercially produced livestock feed. As a result, despite efforts to reduce AGP use in poultry production, a significant challenge is that farmers may unknowingly purchase feed pre-formulated with AGPs. Enhanced transparency in feed formulations is crucial, allowing farmers to make more informed decisions about the feed they purchase. Effective regulatory oversight hinges on the establishment of coherent and well-defined policies. However, the process of policymaking is often complicated by intricate power dynamics [33]. The dominance of hierarchical linkages within vertically integrated poultry producers suggests that efficiency is primarily driven by lead firms, which coordinate activities across the value chain by managing inputs such as animal feed, broiler production, and hygiene standards at abattoirs, while aligning operations with the quality requirements of large retailers and fast-food chains [34]. The analysis highlighted how the actions of these lead firms not only drove value chain efficiency but also influenced policy outcomes, including the regulation of AGP use, often in alignment with industry interests. As a result, achieving meaningful regulatory reform requires not only robust policy frameworks but also navigating the complexities of these power dynamics to ensure that regulations are effectively implemented and enforced.

As consumer awareness of antimicrobial resistance grows, South Africa and other developing countries face the challenge of balancing productivity with responsible antibiotic use by investing in AGP alternatives [35] and strengthening regulatory oversight. The South African Antimicrobial Resistance National Strategy Framework (2018–2024) emphasizes the need for evidence-based prevention, infection control, and optimal use of antimicrobials, aligning with international standards and global health initiatives. Aligning AGP policies with international standards and global health initiatives supports sustainable development and public health goals, ensuring a holistic approach to animal agriculture that benefits all stakeholders [36]. The World Health Organization and the World Organization for Animal Health have issued recommendations that South Africa has incorporated into the development of its national strategies.

## 5 Mechanisms of action of antibiotic growth promoters

Although AGPs have been used in livestock for nearly 60 years, the mechanisms by which they promote growth remain poorly understood. Some of the possible ways in which antibiotics enhance growth include reducing the competition for nutrients by gut microbes, improving nutrient absorption and utilization due to a thinner intestinal wall in animals fed with antimicrobials, preventing disease by suppressing subclinical infections, and lowering the production of microbial metabolites that can hinder growth [15,37]. Additionally, it has been proposed that AGPs may reduce immunologic stress in the intestinal lining [38]. It has also been hypothesized that reducing the bioenergetic costs of gut inflammation explains the positive effects of AGPs on animal efficiency [9]. Other proposed mechanisms include reduction of bacterial metabolites, decreased microbial competition for host nutrients [8], inhibiting the production of toxins in the gut [39], and regulating the host's immunity and inflammation by a direct anti-inflammatory effect [40]. Determining the effects of AGP on microbiota and host response is challenging due to the complex interactions among the microbial community, the host, and the environment. It has, however, been reported that the mechanism of action of antibiotics as growth promoters is most likely linked to their antimicrobial effects [9,16].

**Fig. 1:**
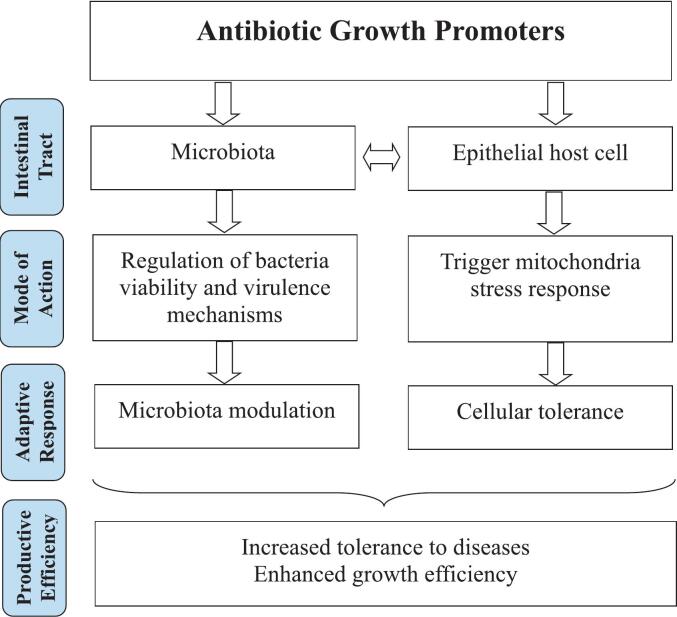
Schematic diagram of the hypothesis effects of antibiotic growth promoters on poultry performance. Adapted from: Miyakawa et al. [9].

Gut microbiota plays a crucial role in the productive efficiency of birds by providing nutrients and energy, maintaining host health, modulating immune responses, and facilitating competitive exclusion of pathogens, particularly in the small intestine [9]. Exposure to different antibiotics, either alone or in combination, can produce synergistic effects in bacteria, regardless of bacterial characteristics or antibiotic type [41]. Many antibiotics can alter bacterial metabolism through different mechanisms of action, influenced not only by the species of the bacteria but also by the characteristics of other surrounding microorganisms [16]. Given the complexity and diversity of the gut microbiota and the possible alterations caused by AGPs, proposing a simple and distinctive explanation for the varied classes of antimicrobials exhibiting growth-promoting effects is difficult [9]. Furthermore, the mode of action of AGPs on the development of infectious diseases cannot be solely attributed to their direct antibacterial properties [42], implying that other AGPs may exert other effects beyond their antimicrobial action. Therefore, the mechanisms of action of AGPs are still unidentified, and explanations on compound-specific mechanisms remain largely elusive [43]. Miyakawa et al. [9] proposed a hypothesis to explain how AGPs could improve productive efficiency in chickens (Fig. 1). In that hypothesis, subtherapeutic levels of antibiotics are thought to modulate microbiota and mitochondrial function, increasing cellular defense mechanisms by inducing an adaptive response.

## 6 Antimicrobial resistance

The overuse of antibiotics in both human and veterinary medicine drives antibiotic resistance [44,45]. Common practices in animal husbandry, such as mass medication (metaphylaxis) and the use of broad-spectrum antibiotics, are often favored over individual treatments due to logistical challenges [20]. Therapeutic antibiotics are typically administered for short durations at doses well above the minimum inhibitory concentration for target bacteria [46,47]. In contrast, AGPs are delivered in feed at lower doses over extended periods [48,49], presenting a lower selection pressure that encourages the development of resistance in target bacteria. Over time, these practices contribute to a bacterial reservoir capable of transferring resistance to pathogens affecting both animals and humans [50]. Consequently, antibiotic resistance has become a critical public health concern and one of the foremost health challenges of the 21st century [51,52].

Poultry treated with antibiotics often harbor significant populations of antibiotic-resistant bacteria, which can be transmitted to humans through environmental exposure, direct contact with animals, or through meat and eggs [17]. This makes antibiotic resistance a quintessential One Health issue, requiring integrated action across human, animal, and environmental health [53]. According to the World Health Organization, One Health is an integrated, unifying approach that aims to sustainably balance and optimize the health of people, animals, and ecosystems [54]. Despite the significant risks posed by antibiotic-resistant bacteria in food, data on microbial quality and safety in intensive poultry production remain limited [55]. South Africa's lack of an effective antibiotic resistance surveillance system represents a major stumbling block that complicates efforts to monitor antimicrobial sales and use. Implementing a robust surveillance program is essential to track and manage antibiotic use across the country [56].

### 6.1 Mechanisms of action

**Fig. 2:**
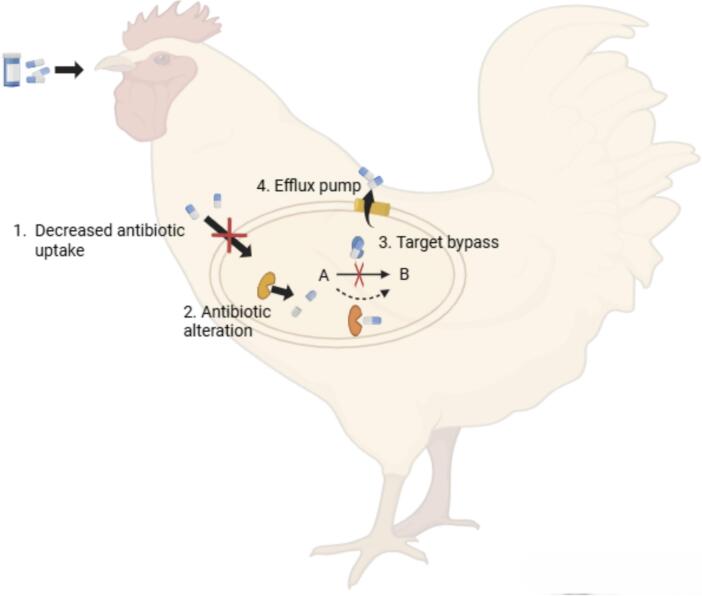
Antibiotic resistance mechanisms in poultry bacterial pathogens. Adapted from: Pulingam et al. [57].

The selection pressure for resistant bacterial strains is increasing due to the usage of antibiotics for therapy and growth promotion. The resultant bacterial resistance occurs via several mechanisms that include antibiotic inactivation, reduced antibiotic penetration, activation of efflux pumps, and target bypass, as illustrated in Fig. 2 [18,57].

#### 6.1.1 Inactivation of antibiotics

Antibiotic inactivation occurs when resistant bacteria produce enzymes that neutralize antibiotic molecules through hydrolysis, group transfer, or redox reactions [18,57,58]. An example of antibiotic inactivation by enzymatic hydrolysis is the production of β-lactamases against penicillins. Additional resistance mechanisms include modification of penicillin-binding proteins, reduced membrane permeability, and increased efflux [59]. However, redox-based inactivation, involving oxidation of the antibiotic molecule, is a less common resistance mechanism [60].

#### 6.1.2 Reduced antibiotic penetration

Given that most antibiotics used in clinical practice have intracellular targets, resistant bacteria have evolved to reduce the permeability of their cell membranes, thereby limiting the penetration of antibiotics. Hydrophilic drugs rely on porin channels to enter microbial cells. However, resistant bacteria often have reduced porin channel concentrations, which slow down drug penetration [57,61]. As a result, the bacterial cell membrane develops an antibiotic permeability barrier, which results in innate resistance to a range of antibiotics [18,57].

#### 6.1.3 Activation of efflux pumps

The membrane of the bacterial cell wall contains transport proteins called efflux pumps, which are responsible for extruding harmful substances from the cellular environment and transporting nutrients [57]. Resistant bacteria have a much more efficient efflux pump system that quickly eliminates antibiotics from the cellular environment before any damage is done [62]. Nonetheless, it is currently acknowledged that the efflux mechanism plays a key role in antibiotic resistance across a wide range of classes. While multimodal efflux pumps can extrude out a range of structurally and functionally distinct antibiotics, efflux pumps can be exclusive to a single antibiotic [57,58,63]. The antibiotic classes that are effluxed are β-lactams, macrolides, fluoroquinolones, fourth-generation carbapems, and cephalosporins [57].

#### 6.1.4 Target bypass

For the cells to become resistant to an antibiotic, bacteria create an alternative pathway to bypass the antibiotic by making the primary target redundant [57,58,61,63]. For this to occur, the original antibiotic may not effectively impede the acquisition of a different gene that can provide the cell with the necessary characteristics.

### 6.2 Transmission of resistance: poultry to humans

**Fig. 3:**
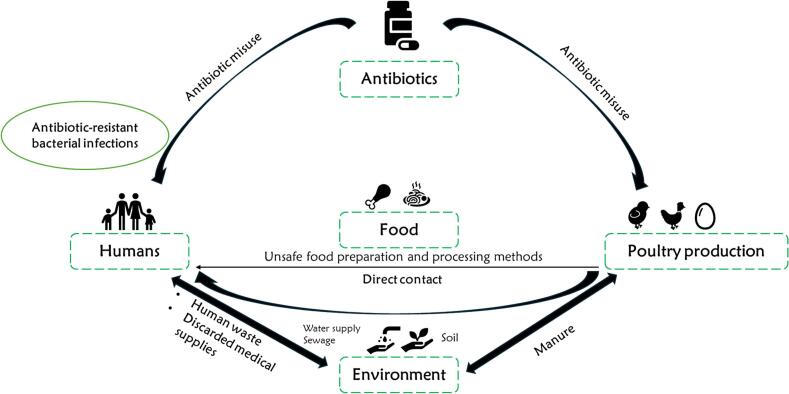
Transmission pathways of antimicrobial-resistant bacteria from poultry to humans. Adapted from: Ahmad et al. [67].

Some antibiotics used as AGPs in poultry, like oxytetracycline, aminoglycosides, and penicillin, are also used to treat bacterial infections in humans [62]. Recent reports show that several antimicrobials classified as critically important for human medicine, often reserved as last-resort treatments for resistant infections, have relatively low sales volumes in human healthcare [64]. To preserve their efficacy, the WHO recommends restricting the use of these antibiotics in food-producing poultry [65]. Excessive use of these drugs in animals, coupled with poor adherence to withdrawal periods, results in antibiotic residues in animal products that pose significant health risks to humans [47,66]. This practice also promotes the development and dissemination of antibiotic-resistant bacteria, which can be transmitted to humans via meat consumption, contact with contaminated manure [62,66], and other exposure routes (Fig. 3).

Multiple bacterial species affecting poultry, including *Staphylococcus*, *Salmonella*, *Escherichia coli*, *Klebsiella*, *Shigella*, *Enterobacter*, *Citrobacter*, *Serratia*, *Proteus*, *Helicobacter*, and *Pasteurella* [[68], [69], [70]], have demonstrated resistance to a broad spectrum of antimicrobials, such as ampicillin, neomycin, polymyxin B, tetracycline, ciprofloxacin, gentamicin, erythromycin, fluoroquinolones, cephalosporins, sulphonamides, and macrolides [[71], [72], [73], [74]]. In response, the World Health Organization recommends that national regulatory bodies across the agriculture, veterinary, and pharmaceutical sectors prohibit the use of antimicrobials as growth promoters [75]. Furthermore, administration of third- and fourth-generation cephalosporins and fluoroquinolones in food animals should be restricted to cases with veterinary prescription and justified clinical indication [76]. Therapeutic use of narrow-spectrum antibiotics must be guided by clinical evidence and antimicrobial susceptibility testing. National guidelines for prudent antibiotic use in food animals should be developed and enforced by veterinary authorities.

## 7 Regulatory frameworks for antibiotic use in animals

### 7.1 South Africa's regulatory framework

In South Africa, legislation governing antibiotic use in animals is still lenient. Their use is regulated under the Fertilizers, Farm Feeds, Agricultural Remedies, and Stock Remedies Act, 1947 (Act No. 36 of 1947), administered by the Department of Agriculture, Forestry and Fisheries; and the Medicines and Related Substances Control Act (Act 101 of 1965), administered by the National Department of Health (NDoH). Antibiotics intended for use by non-professionals, primarily farmers, are authorized under Act 36 as stock remedies and are accessible without a prescription [24]. As a result, farmers routinely use AGPs as part of livestock management practices. The veterinary medicine regulations in South Africa present notable shortcomings compared to global best practices outlined by the World Health Organization (WHO). Of particular concern is the dual system of regulation, where antibiotics are subject to different standards depending on whether they are classified as stock remedies under Act 36 or fall under Act 101. While Act 101 mandates that antibiotics adhere to stringent manufacturing requirements, Act 36 lacks similar provisions, leading to inconsistencies in product quality and safety [24]. These regulatory shortcomings have notable implications, particularly regarding the use of antibiotics for growth promotion in livestock. Without stringent manufacturing requirements and professional oversight, there is a risk of overuse and misuse of antibiotics in animal agriculture, contributing to the development of antibiotic-resistant bacteria. This misuse not only compromises animal welfare but also poses significant public health risks, as antibiotic-resistant pathogens can spread through the food chain and environment, affecting human health.

Despite the more lenient overall regulations compared to regions like the EU, South Africa has implemented measures that reflect efforts to balance agricultural productivity with the need to mitigate the risk of antibiotic resistance and protect public health. Such measures include the establishment of withdrawal periods for antibiotics in poultry and other livestock, ensuring that any antibiotic residues are eliminated from the animals before they enter the food chain [19]. This serves to minimize the risk of antibiotic residues in meat and poultry products, addressing public health concerns. However, while these measures exist, their effectiveness is sometimes compromised. Not all farmers consistently adhere to withdrawal periods, which can lead to antibiotic residues in food products [77]. This inconsistency in following best practices undermines efforts to control antibiotic resistance and poses a significant risk to public health. This not only risks public health but also threatens South Africa's reputation in the global market for meat products. It goes without saying that countries with stringent AGP regulations may restrict imports of animal products from nations with less stringent controls, potentially affecting South Africa's export potential. For example, Article 118 of Regulation (EU) 2019/6 on veterinary medicinal products, which came into effect on January 28, 2022, mandates that any products imported into the European Union must originate from animals that have not been treated with growth-promoting antibiotics. Moreover, in recent years, consumers have become more health-conscious and are driving an ever-expanding market for meat raised without antibiotics [78].

### 7.2 Antimicrobial monitoring in South Africa

South Africa still needs to align with international standards to enhance the quality and safety of products, to make them more competitive in global markets. Locally, public health still needs to be prioritized, aligning with global agendas including Sustainable Development Goal (SDG) 3 (good health and well-being), SDG 12 (responsible consumption and production), and the one health approach, which emphasizes the interconnectedness of human, animal, and environmental health.

**Fig. 4:**
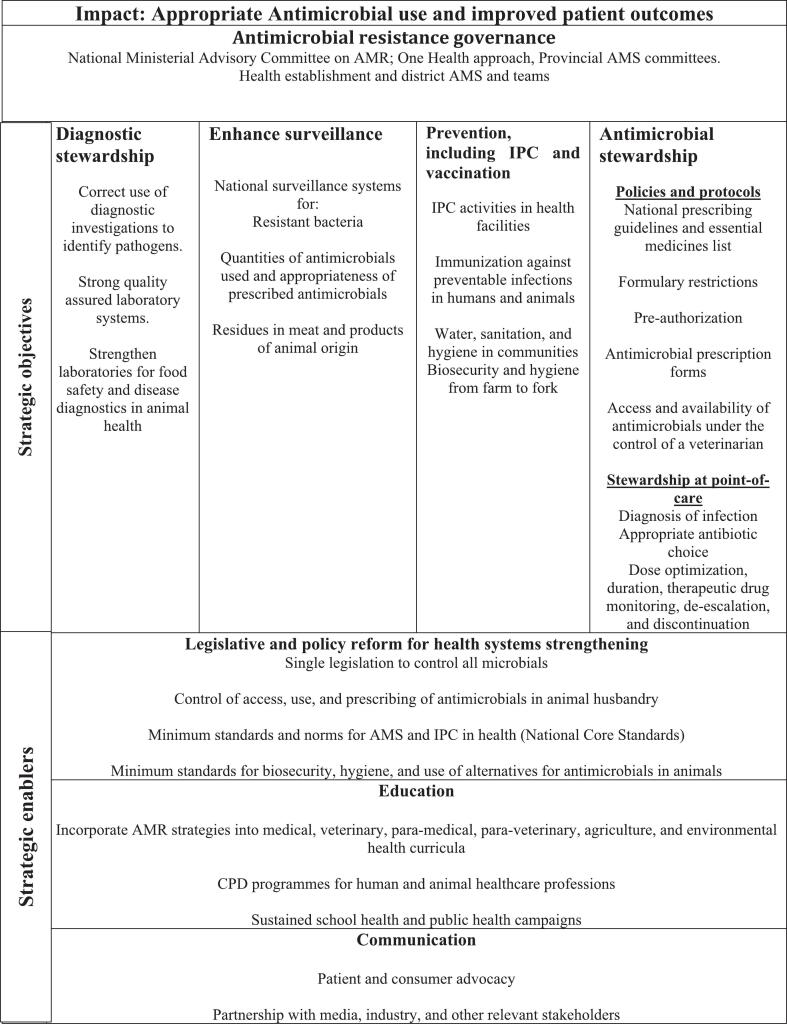
A top-down One Health governance framework for the National Antimicrobial Resistance Strategy, led by the National Department of Health (South Africa) in collaboration with human, animal, and environmental health sectors. Adapted from: NDoH [80].

Currently, the Department of Agriculture, Forestry and Fisheries (DAFF), in partnership with the South African Animal Health Association (SAAHA), is responsible for actively monitoring and reporting the use of antimicrobials in animals. This reporting is done in accordance with the requirements set by the World Organization for Animal Health (OIE) [79]. Aligning with OIE standards ensures that South Africa can benchmark its antimicrobial usage data against international standards, allowing for continuous improvement and better decision-making in the agriculture sector. As part of its commitment to the global fight against AMR, South Africa pledged to develop a National Action Plan (AMR). By October 2014, the Antimicrobial Resistance National Strategic Framework 2014–2024 was created and launched, with support from key stakeholders in human and animal health, agriculture, and science and technology [80]. The implementation of the Strategic Framework (Fig. 4) was led by the National Department of Health. The Framework includes contributions from various entities such as the Department of Agriculture, Forestry and Fisheries (DAFF), the Department of Science and Technology, laboratory services, and clinician bodies. It also incorporates civil organizations like the Treatment Action Campaign (TAC) and Médecins Sans Frontières. Additionally, regulatory agencies are involved, including the South African Veterinary Council (SAVC), which oversees veterinary practice in South Africa, and the Medicines Control Council (MCC) [81]. The Framework outlines South Africa's strategy for managing antimicrobial resistance, aiming to prevent the rise of resistant microbial infections, enhance patient outcomes, and improve livestock health and production. The framework reflects a top-down One Health governance model, with the National Department of Health at the helm, coordinating multi-sectoral efforts across animal, human, and environmental health sectors to address antimicrobial resistance through surveillance, stewardship, prevention, and public awareness. A key component of the framework is surveillance, which entails gathering data to support research, strategic initiatives, and policy and planning decisions. However, challenges persist in the availability of reliable data, system integration, and resource allocation. Data on the actual supply and consumption of volumes of antimicrobials utilized in animal health are very scant, and there is a lack of information about the volumes and patterns of antimicrobial usage in food animals [1]. To strengthen the effectiveness of this framework, it is essential to focus on enhancing data reliability, integrating health information systems, upgrading infrastructure, training personnel, promoting cross-sector collaboration, engaging communities, and supporting policy coherence.

### 7.3 The way forward for South Africa

Addressing the use of antibiotics within South Africa's regulatory framework requires a balanced approach that considers the needs and challenges of all stakeholders. Banning AGPs may not be the ultimate solution at present, especially without any viable alternatives. Instead, a systems approach that includes comprehensive regulations, stakeholder engagement, and sustainable practices can help mitigate the risks of antibiotic resistance while ensuring the competitiveness and viability of South Africa's livestock industry. Additionally, learning from countries that implemented bans can provide strategic insights for decision-making. For example, studies conducted in Denmark and Sweden, along with recent estimates from the United States, indicate that phasing out AGPs may have limited negative economic effects. However, these findings may not be universally applicable to every country or every operation within a country. Countries with advanced production systems that adhere to high standards of hygiene and production practices are likely to experience minimal impacts on productivity and economic performance when AGPs are withdrawn. This suggests that the impact of phasing out AGPs can vary significantly based on the existing practices and infrastructure within different agricultural systems [82].

## 8 Monitoring antibiotics in the poultry food chain

### 8.1 Antibiotic residues in animal-derived products

The safety of animal-derived products has taken center stage in recent times. Continuous use of antibiotics for therapeutic and subtherapeutic purposes during poultry rearing can result in significant residues in tissues and blood. The presence of antibiotic residues in foods occurs when farmers extract food from animals treated with antibiotics without adhering to the prescribed withdrawal [83,84]. The consumption of food products with antibiotic residues has been reported to result in bone marrow dysfunction, birth defects, and increased risk of genetic mutations [85]. The Foodstuffs, Cosmetics and Disinfectants Act (Act 54 of 1972) in SA provides for the monitoring of antibiotic residues in the food chain. The act incorporates regulations on the acceptable levels of antibiotic residues in food to ensure that food is safe for human consumption. In addition, the Animal Disease Act (Act 35 of 1984) regulates the use of veterinary medicine, such as antibiotics, to reduce their residues in animal products. Furthermore, the Department of Agriculture, Land Reform and Rural Development (DALRRD) has a program called the National Residue Monitoring Programme (NRMP) for monitoring and controlling the levels of antibiotic residues in animal-derived food products. While SA's regulatory framework is robust and comparable to international standards, the EU and US have more detailed monitoring and reporting systems. Even though SA's regulations are aligned with international standards such as Codex Alimentarius to ensure food safety, there is a need for more frequent testing and stricter enforcement mechanisms. There is a need for comprehensive surveillance and greater awareness among stakeholders in the food chain [86]. This should include targeted monitoring of antimicrobial residues at various points in the poultry production chain, including farms, slaughterhouses, and retail outlets. Enforcement can be improved through regular inspections, the application of penalties for non-compliance, and the use of traceability systems to monitor antibiotic use. Additionally, stakeholder education is crucial, particularly for farmers, animal health practitioners, feed suppliers, and consumers, to promote prudent antibiotic use, observance of withdrawal periods, and better animal management practices that reduce reliance on antibiotics.

The regulations governing maximum residue limits (MRLs) for veterinary medicines in South Africa are influenced by international standards set by the Food and Agriculture Organization (FAO) and the European Union (EU). The Foodstuffs, Cosmetics and Disinfectants Act (Act No. 54 of 1972) specifies the regulations for MRLs of veterinary medicines in foodstuffs in South Africa. However, SA does not have set standard limits.

These regulations safeguard the levels of antibiotic residues in animal-derived food products to ensure that they are within safe limits to protect consumer health. Furthermore, the Codex Alimentarius Commission also ensures international food standards, including MRLs for veterinary drugs, are used globally to ensure food safety and expedite international trade (Table 1). Several studies have also been conducted to identify common antibiotic residues found in animal products (Table 2).

**Table 1:** Maximum residue limits (MRLs) in animal products.

Product	Antibiotic residue	MRL (μg/kg)
Liver	Tetracyclines	200
Meat	Tetracyclines	600
Kidneys	Tetracyclines	1200
Liver	Enrofloxacin and Ciprofloxacin	200
Meat	Enrofloxacin and Ciprofloxacin	100
Kidney	Enrofloxacin and Ciprofloxacin	300

Adapted from: FAO [87].

**Table 2:** Common antibiotic residues found in poultry products.

Product	Antibiotic residue	References
Eggs	Sulfonamides, quinolones, macrolides, b-lactams	[88,89]
Chicken meat	Tetracyclines, benzimidazoles, enrofloxacin, fluoroquinolones, ciprofloxacin	[90,91]

Ciprofloxacin levels of 600 μg/kg (14.2 and 1280 μg/kg) were found in chicken breast meat and liver samples in a South African study [91], providing some evidence that antibiotic residues can pose a health challenge for consumers of animal products. Given that antibiotic residues in poultry products represent a food safety breach, a robust monitoring system is critical to mitigate public health risk. An effective monitoring system should be supported by sufficient regulatory provisions and sound detection methods for antibiotic residues in poultry products.

### 8.2 Detection methods

The methods used to determine antibiotic residues from animal-derived products can be classified as either confirmatory or screening procedures. The screening procedures used include enzyme-linked immunosorbent assay (ELISA), fluoroimmunoassay (FIA), time-resolved fluoroimmunoassay (TRFIA), biosensors, and surface-enhanced Raman spectroscopy (SERS) [[92], [93], [94]]. Whilst the confirmatory procedures are liquid chromatography mass spectrometry (LC-MS), liquid chromatography with ultraviolet (UV), ultra-high performance liquid chromatography (UHPLC-MS/MS), high performance liquid chromatography-mass spectrometry (HPLC-MS/MS), and capillary electrophoresis (CE) [95,96]. It should be noted that the confirmatory methods are quite costly, labor-intensive, time-consuming, and require very skilled personnel and sophisticated laboratories [94]. Despite these challenges, they are still the most widely used methods for the determination of antibiotic residues in animal-derived products.

Screening methods involve both qualitative and semi-qualitative microbiological and immunoassays. The screening methods are less complicated to use, less costly, and reliable. However, they are time-consuming due to the extended incubation time and can lack specificity [93]. In the food industry, the use of fully automatic biosensors, such as electrochemical, optical, and mass-sensitive biosensors, has rapidly increased. Biosensors are rapid and enable specific recognition of present antibiotics [96,97]. In South Africa, the common methods used for screening are ELISA, HPLC, and TLC [80,91,98].

## 9 Prospects for phasing out antibiotic growth promoters

### 9.1 AGP-free poultry: balancing public health risks and financial gains

A study by Graham et al. [99] was conducted to analyze the economic impact of AGPs in food animal production in the US context, using data and examples relevant to American agricultural practices and regulations. The study focused on the financial benefits of AGPs in terms of improved feed efficiency and growth rates. While they found that AGPs provide some economic advantages by lowering production costs, the overall benefit was relatively small, especially considering the potential public health risks linked to antibiotic resistance. The study estimated a net loss of $0.0093 per chicken, with savings from AGP use outweighing the decrease in production. However, the analysis did not account for veterinary costs or the potential impact of increased variability in broiler weights. The authors also argued that the short-term cost savings may not be worth the long-term health risks, including rising antibiotic resistance in humans, which could result in higher medical costs and reduced antibiotic efficacy. Van Boeckel et al. [2] assessed the economic benefits of AGPs in the livestock sector, investigating their effects on production efficiency, feed conversion rates, and profitability in poultry, pig, and cattle farming. Using global data, the study showed that AGPs improve growth rates and reduce feed costs, contributing to higher economic returns, particularly in intensive farming systems. However, the study also highlighted concerns about AMR linked to AGP use, suggesting that although AGPs offer short-term economic gains, their long-term public health risks could outweigh these benefits.

### 9.2 Lessons from developing countries

Developing countries remain hesitant to phase out antibiotic growth promoters, yet doing so is essential to reduce antimicrobial resistance and safeguard both human and animal health from multidrug-resistant pathogens. However, the potential impacts of this action have not been fully examined in these countries, while several studies have been carried out in developed countries that have already banned or restricted AGP use in food animals. In theory, phasing out AGPs should cause negative economic impacts in poultry production, as they play a crucial role in ameliorating stress-induced maladies in intensive production systems, thereby enhancing productivity. Recent estimates of the potential economic impact of a ban on AGPs are limited to a few countries such as the United States (US), and some EU countries [82]. In the US, the USDA estimates of the market-level effects of a ban on AGPs in the broiler production indicated limited effects. The USDA estimated that broiler production would decrease by, at most, 1.12 %, and as a result, wholesale prices would rise by less than 1 % to a maximum of 2.6 %, while total production costs would increase by 1.45 %. Likewise, MacDonald and Wang [100] demonstrated that suspending AGPs would have no statistically significant impact on broiler production operations, after accounting for other factors influencing production such as labor, capital, and additional inputs. However, they also found that producers who did not use AGPs before the ban could see a 2.1 % increase in production value, while those who previously relied on AGPs might experience either a loss or a gain. The higher fees paid by integrators are likely to compensate producers for the additional costs of production without AGPs. Integrators often contract with independent farmers, paying them to raise the animals according to specific guidelines. They supply the birds, feed and sometimes other resources, while the farmer provides labor, housing, and day-to-day animal care. The integrators then pay the farmers based on production outcomes, often with bonuses or higher fees for meeting standards, such as raising chickens without AGPs. In Denmark's broiler industry, the ban on AGPs between 1995 and 1999 had no impact on mortality rates, average weight gain, or productivity (measured as kg of broilers produced per m^2^ per production) [101]. The estimated net cost increase from removing AGPs in poultry production was EUR 0.00, indicating that the slight reduction in feed efficiency was at least partially offset by the savings from no longer purchasing AGPs [102].

The U.S. and Danish studies report minimal market impacts following AGP bans. However, such outcomes may not extrapolate to other contexts, where differences in production scale, infrastructure, and management could amplify economic effects. According to Laxminarayan et al [82], countries with modern production systems that implement good hygiene and production practices are likely to experience minimal impacts on productivity and economic outcomes from phasing out AGPs. In contrast, countries with less optimized production systems may face more significant productivity losses, leading to greater economic effects. This could be the case for developing countries such as South Africa. Furthermore, in the US, MacDonald and Wang [100], reported that a ban on AGPs would impact producers differentially, according to location, farm size, production practices, species, and stage of production. Moreover, in studies describing the Swedish experience after that country's 1986 AGP ban, it was reported that the effect of a ban would also depend on management variables and health and sanitation practices [103].

### 9.3 Potential impacts of phasing out AGPs in South Africa and similar countries

Transitioning to AGP-free production in subtropical environments presents significant challenges due to higher baseline disease pressures. Unlike temperate regions, where AGP-free systems are more viable due to lower pathogen loads and stable environmental conditions [14], subtropical climates are characterized by higher temperatures and humidity. These conditions promote the proliferation of pathogens such as *Escherichia coli*, *Salmonella*, and *Clostridium perfringens*, increasing the prevalence of diseases like colibacillosis, coccidiosis, and necrotic enteritis [104,105]. Heat stress, which is more severe in these climates, further compromises poultry immune function, exacerbates oxidative stress, and worsens gut health challenges [106]. Suboptimal biosecurity practices, especially in smallholder and informal systems with high bird densities, further compound the risk of disease outbreaks. In contrast, temperate regions often benefit from more intensive, technologically advanced operations with better biosecurity protocols [107,108]. Therefore, the combination of climate-related stressors and limited infrastructure in developing regions contributes to greater reliance on antimicrobial interventions, making the shift to AGP-free production systems more complex. To mitigate these challenges, producers must adopt a range of strategies, including robust biosecurity, nutritional interventions, and improved management practices [15,109]. However, these adaptations require significant investment, training, and supportive policies.

Estimating the full economic impact of an AGP ban in SA and other developing countries is challenging due to limited data availability. Poultry producers currently benefit from AGPs through enhanced performance and health, improved health outcomes, and reduced production risks. Small-scale and emerging farmers are likely to be the most affected by the ban, as they often lack the financial and technical capacity to adopt alternative approaches. The operations typically face challenges such as low hygiene standards and minimal biosecurity, leaving them more vulnerable to production losses. Conversely, large-scale, modern producers may be better positioned to absorb additional costs or transfer them to consumers, owing to better infrastructure and disease control practices. Insights from the United States, such as those reported by Sneeringer et al. [110], suggest that banning AGPs can lead to increased production costs, higher feed expenses, and a rise in disease incidences. This is because AGPs play a crucial role in maintaining gut health and feed efficiency, reducing the need for high-cost feed formulations and therapeutic interventions. Without AGPs, producers may need to rely on alternative additives, enhanced hygiene protocols, and veterinary services, all of which can significantly raise production costs. Consequently, the price of poultry products may increase, potentially reducing competitiveness in both local and export markets. Similarly, a meta-analysis by Cardinal et al. [111] found that removing AGPs from broiler diets resulted in higher production costs and higher market prices.

In a country like SA, where food insecurity is already a concern for many, rising poultry prices could have serious social and economic consequences. Low-income households, which spend a significant proportion of their income on food, would be disproportionately affected. Reduced access to affordable animal protein could negatively affect nutrition and health outcomes, particularly in vulnerable populations [112]. Despite the short-term economic concerns, the long-term public health and environmental benefits of phasing out AGPs could outweigh the initial challenges. As demonstrated in other countries, the agricultural sector can adapt over time, but doing so requires investment in innovation, research, and supportive policies from the government.

One promising avenue is the adoption of nutraceuticals, which are bioactive compounds that positively enhance animal health, immunity, and productivity. Nutraceuticals can enhance digestive enzyme activity, restore microbial balance, and stimulate gene expression related to nutrient transport, metabolism, and immunity [113]. These include probiotics, prebiotics, enzymes, organic acids, phytochemicals, and various botanical products [19,25,113,114]. While they may not provide the same immediate growth benefits as AGPs, their long-term benefits make them a sustainable alternative. In SA, emerging research is already exploring the efficacy of these alternatives. Notable studies have evaluated organic acids [115]; a combination of live *Bacillus subtilis* probiotic, with benzoic and fumaric acids, protease enzymes, and chelated minerals [116]; *Moringa oleifera* leaf meal in comparison with probiotics and organic acids [25]; and in-feed encapsulated *Salmonella*-specific bacteriophages [117]. Although results have been inconsistent, this growing body of work reflects a national movement towards antibiotic-free poultry production systems. To ensure success, future antimicrobial reduction efforts in SA should focus on expanding access to viable AGP alternatives, particularly for small-scale and emerging farmers. This will require targeted investments, policy support, public-private partnerships, and farmer training programs aimed at sustainable production.

## 10 Conclusions

This review identified the stumbling blocks for transitioning to AGP-free systems in developing countries such as South Africa, such as a lack of sustainable alternatives, higher disease burdens in tropical and subtropical regions, lower biosecurity, and inadequate regulatory and monitoring frameworks. Several strategies to address these challenges were reviewed, and these could push and pull all stakeholders towards the objective of AGP-free poultry production. These could include alternative policies, practices, technologies, and antimicrobials that could be exploited to drive the transition to AGP-free poultry production. A review of existing regulation and policy frameworks in South Africa and other developing countries indicated that these may be insufficient to drive the transition to AGP-free poultry production. Carefully crafted regulations and policies can create an enabling environment that promotes the shift to AGP-free poultry production. These could provide a framework for strategies such as the implementation of gradual AGP bans/restrictions, creating incentives, setting biosecurity standards, subsidizing AGP alternatives, creating a certification program and market incentives, and promoting research and innovation. In conclusion, the prospects for AGP-free poultry production at present are good but require sustained efforts to create an enabling environment by all stakeholders.

## CRediT authorship contribution statement

**Xola Nduku:** Writing – review & editing, Writing – original draft, Visualization, Conceptualization. **Thuthuzelwa Stempa:** Writing – review & editing. **Nobuhle Sharon Lungu:** Writing – review & editing. **Tandile Nwabisa Ndobeni:** Writing – review & editing. **Victor Mlambo:** Writing – review & editing, Validation, Conceptualization.

## Funding

No funding was received for the preparation of this review paper.

## Declaration of competing interest

The authors declare no conflicts of interest.

## Data Availability

No data was used for the research described in the article.
